# The Evolutionary Origin of Man Can Be Traced in the Layers of Defunct Ancestral Alpha Satellites Flanking the Active Centromeres of Human Chromosomes

**DOI:** 10.1371/journal.pgen.1000641

**Published:** 2009-09-11

**Authors:** Valery A. Shepelev, Alexander A. Alexandrov, Yuri B. Yurov, Ivan A. Alexandrov

**Affiliations:** 1 Institute of Molecular Genetics, Russian Academy of Sciences, Moscow, Russia; 2 Mental Health Research Centre, Russian Academy of Medical Sciences, Moscow, Russia; Emory University School of Medicine, United States of America

## Abstract

Alpha satellite domains that currently function as centromeres of human chromosomes are flanked by layers of older alpha satellite, thought to contain dead centromeres of primate progenitors, which lost their function and the ability to homogenize satellite repeats, upon appearance of a new centromere. Using cladistic analysis of alpha satellite monomers, we elucidated complete layer patterns on chromosomes 8, 17, and X and related them to each other and to primate alpha satellites. We show that discrete and chronologically ordered alpha satellite layers are partially symmetrical around an active centromere and their succession is partially shared in non-homologous chromosomes. The layer structure forms a visual representation of the human evolutionary lineage with layers corresponding to ancestors of living primates and to entirely fossil taxa. Surprisingly, phylogenetic comparisons suggest that alpha satellite arrays went through periods of unusual hypermutability after they became “dead” centromeres. The layer structure supports a model of centromere evolution where new variants of a satellite repeat expanded periodically in the genome by rounds of inter-chromosomal transfer/amplification. Each wave of expansion covered all or many chromosomes and corresponded to a new primate taxon. Complete elucidation of the alpha satellite phylogenetic record would give a unique opportunity to number and locate the positions of major extinct taxa in relation to human ancestors shared with extant primates. If applicable to other satellites in non-primate taxa, analysis of centromeric layers could become an invaluable tool for phylogenetic studies.

## Author Summary

The primate centromere evolves by amplification of alpha satellite sequences in its inner core, which expands and moves the peripheral sequences sideways, forming layers of different age in the “pericentromeric” area. The expanding centromere model poses two main questions: (1) whether the succession of layers is symmetrical on both sides of the centromere, and (2) whether different chromosomes share the same layers. We have analyzed and dated the layers on both sides of human chromosomes 8, 17, and X and shown that they were largely symmetrical on one chromosome and largely shared and arranged similarly in non-homologous chromosomes. The layer pattern revealed that genome-wide waves of expansion of new satellite variants have occurred repeatedly in the human evolutionary lineage. The layers which are likely to be the relic centromeres of our common ancestors with primate taxa follow each other in chronological order. The two layers that do not match any living primate indicate the two completely extinct ancestral taxa aged 26–40 and 18–23 million years. These could be Propliopithecidae (Cathopitecus and Egyptopithecus) and Pliopithecidae (Proconsul), aged 33–35 and 17–27 million years, respectively. The possibility to reveal and date extinct ancestors makes the analysis of satellite layers a unique tool for the reconstruction of primate phylogeny.

## Introduction

Active human centromeres are made of great ape-specific alpha satellite DNA (AS), comprised of ∼171 bp tandem monomers forming nearly identical higher order repeats (HORs) and represented by the “new” suprachromosomal families (SFs) 1, 2 and 3. They are surrounded by much less homogeneous HOR-free “monomeric” AS (SF4 and SF5) often disrupted by transposon insertions [1],[2]. SF4 is usually composed of a single M1 class of monomers with no evidence of higher-order periodicities. SF5 is formed by two types of monomers, R1 and R2, alternating irregularly. R2 is similar to M1 (class A), and R1 represents the first appearance of novel class B monomers, which bind CENP-B protein and presumably have invaded the A-arrays before the great ape divergence [1]. High identity and high copy number of HORs are presumably maintained by an active process called homogenization, which is driven by homologous recombination mechanisms such as unequal crossover and/or gene conversion. The monomeric AS is older than the HOR arrays and resembles AS of lower primates [1]. Divergence patterns and transposon distribution suggest that the “old” domains were once homogenous, but at some point homogenization had stopped and accumulation of sequence divergence and of transposable elements commenced [2]. Thus, old AS arrays are likely the remnants of the centromeres of our primate phylogenetic ancestors, once active and homogenous, but obsolete and degrading since centromeric function and homogenization have shifted to the new AS [1],[2]. Furthermore, analysis of the human X chromosome short arm (Xp) pericentromeric region, the first one sequenced in its entirety, has revealed an age gradient, with most distal Xp AS domain dating to early primate evolution, the HOR domain to the time of great ape divergence and the domains in between being of interim age [3],[4]. Assuming that the succession of AS layers on the long arm (Xq) side is symmetrical, it was proposed that the primate X chromosome centromere “evolved through repeated expansion events involving the central functional AS domain, such that ancestral centromeric sequences were split and displaced distally onto each arm” [4].

Previously, we proposed the existence of a kinetochore-associated recombination machine (KARM) that homogenizes only the active centromere, a model that accounts well for the above observations [1],[2]. Accumulating evidence suggests that topoisomerase II, a DNA decatenating enzyme, is an important part of this machine. In mitosis, it resides in the kinetochore [5]–[7] and plays a crucial role in resolution of the recently discovered chromatin PICH threads that connect chromatid centromeres [8]–[12]. The enzyme introduces double strand breaks into human AS arrays [13]–[15], and in dicentric chromosomes its activity is observed only in the active centromere [16]. As topoisomerase II breaks are known to initiate homologous recombination [17]–[19], the enzyme is a likely candidate for KARM function.

Here we present a complete analysis of AS layers of chromosomes 8, 17 and X and for the first time provide comprehensive comparisons of the entire layer patterns on both arms of one chromosome and between different chromosomes. As expected, the succession of multiple layers appears to be largely symmetrical around the centromere. More surprisingly, the layer structure is to a large extent shared between non-homologous chromosomes, supporting a model of genome-wide expansion events that give rise to new centromeres on many chromosomes within an evolutionary short period of time. Primate comparisons reveal that each major taxon in the human lineage corresponds to a separate “suprachromosomal” centromeric layer, providing a complete record of human ancestry. Comparisons of inter- and intra-species divergence within a layer suggest that, after relocation of the centromere, the dead arrays experienced an unusual burst of mutability. Highly informative structure and their potential role in “centromeric speciation” [20],[21] should make centromeric layers extremely useful for phylogenetic analysis.

## Results

### Analysis of AS in chromosomes 8, 17, and X

**Figure 1:**
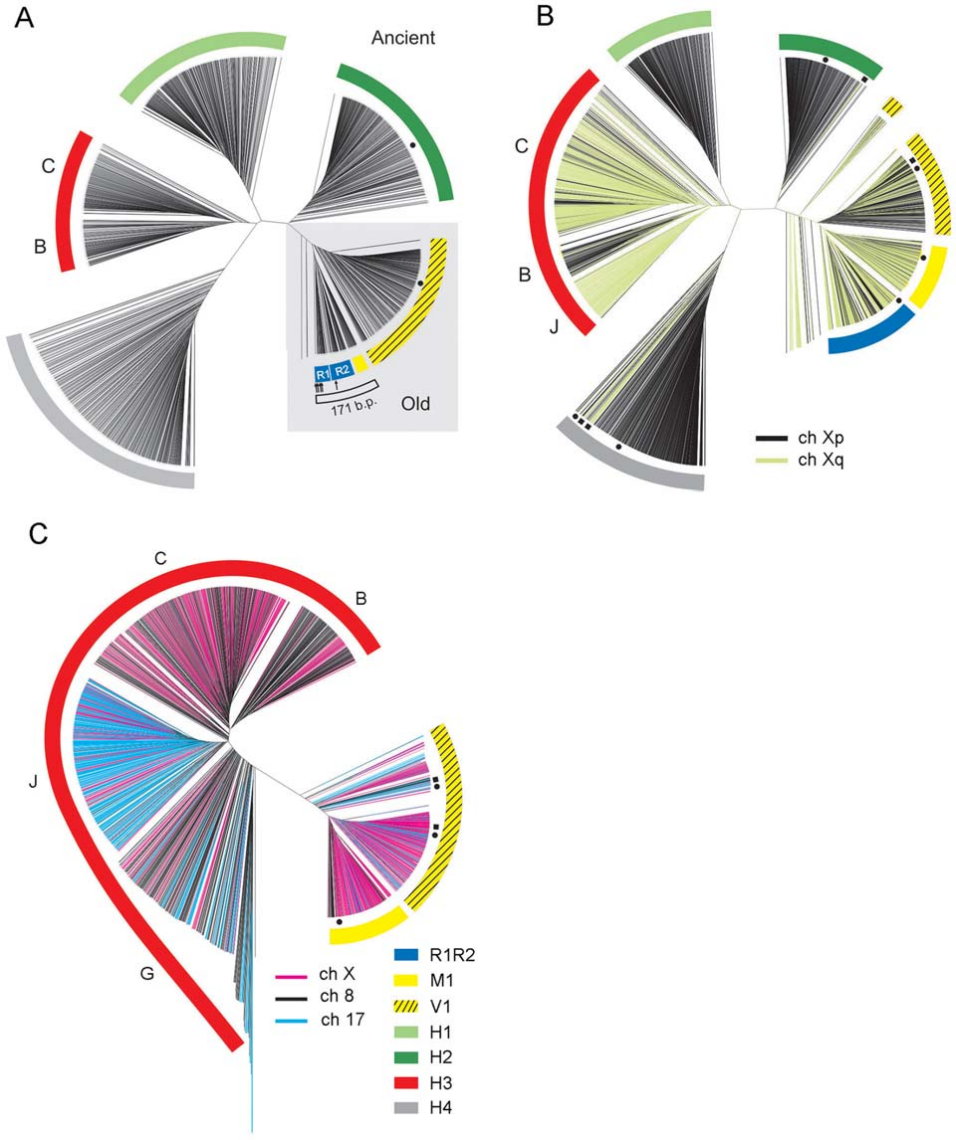
Phylogenetic trees of AS monomers in human chromosomes 8, 17, and X. Each terminal branch represents an AS monomer. The major branches marked by colored arches correspond to colored AS arrays in Figure 2 and monomeric types in Table 1. The positions of runaway monomers that appear in a “wrong” cluster are indicated by dots (1–3 monomers) or squares (4–15 monomers). (A) 1,434 monomers of Xp pericentromeric region plus W1–W5 consensus monomers representing the current SF3 centromere (indicated by arrows, cluster with R1R2). “Old” clades are highlighted by a grey box. All other clades belong to the “ancient” group. The 171 bp AS clade is indicated by an open arch. Branches formed by R1 and R2 monomers are indicated. (B) Monomers of Xq pericentromeric region were added to those shown in (A) (2,516 monomers total). Xp monomers are shown in black and Xq monomers in khaki. No major new clades appear. Grey, olive, and green clades are Xp-specific, except for a few runaway monomers. In blue, yellow, and yellow-striped branches, the monomers from both arms of X chromosome are well-mixed. (C) Phylogenetic tree of the red, yellow-striped, and yellow monomers from chromosomes 8, 17, and X (2,588 monomers). Due to a large number of red monomers in chromosome 8, every 5^th^ 8p and every 4^th^ 8q red monomer were taken into analysis. Monomers from different chromosomes (black, blue, and purple for 8, 17, and X, respectively) mix well on the tree. Subclades in the red branch are indicated by letters B, C, J, and G above the red arch.

**Figure 2:**
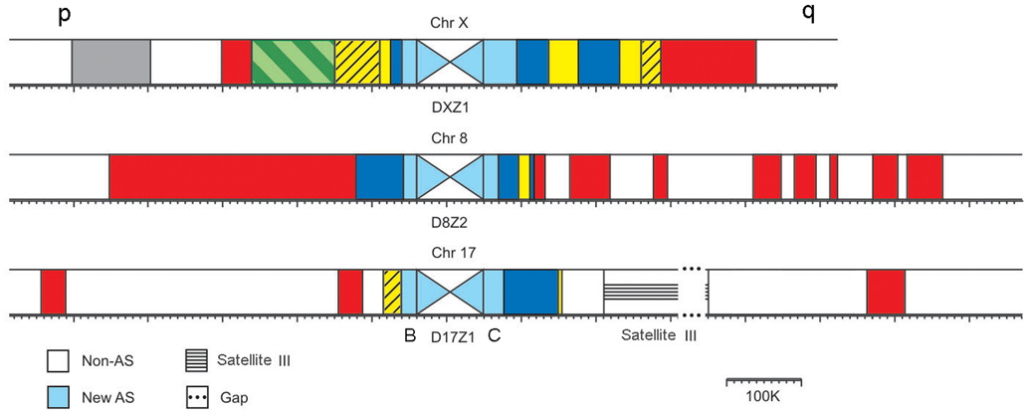
Alpha satellite layers in human chromosomes 8, 17, and X. Each colored domain represents an AS array composed of monomers that belong to the same branch on phylogenetic trees shown in Figure 1. Chromosome domains and the arches marking different branches are in the same colors in Figures 1 and 2. Colored layers are partially symmetrical around the centromere on one chromosome and partially shared between different chromosomes. The p and q arms of the chromosomes are indicated. The diagonally crossed white and light blue central boxes represent the new AS HOR domains, which form current centromeres. They are shown not to scale. For chromosome 17, we show the presumed organization of the HOR domain. The central D17Z1 16-mer HOR array is flanked by two homogenous 14-mer HOR arrays, D17Z1-B on the p arm [24], and a distinct one termed D17Z1-C on the q arm (see Text S1 for details).

In human chromosomes 8, 17 and X the pericentromeric regions of both chromosome arms have been sequenced almost completely, starting from the surrounding euchromatic regions and into the HOR arrays that constitute current centromeres. We used the genomic builds of these chromosomes (see Table S1 for reference sequences) to identify and extract all AS monomers and analyzed them using a cladistic approach [3], [4], [22]–[24] based on construction of monomeric phylogenetic trees (Figure 1; see Text S1 for details). This resulted in identification of a number of distinct AS domains in each centromere (listed in Table 1 and shown in different colors in Figure 2). The main criteria to assign differently located arrays to the same-color suprachromosomal layer was their structural similarity and ability to “mix well” on phylogenetic trees with each other, but not with the other layers. Our results do not contradict previous partial analysis of AS on these chromosomes [3],[4],[24] and throughout the genome [1],[2]. However, a few important new features were noted (Table 1 and Text S1) and the entire complex pattern of AS relationships was revealed for the first time.

**Table 1:** Structural features in dead AS layers.

Layer	Monomer length (bp)	Monomer types*	Arrangement	Age group**
Blue	171	R1 (B), R2 (A)	irregular	old
Yellow	171	M1 (A)	monomeric	old
Yellow-striped	172	V1 (A)	monomeric	old
Olive-green	172	H1 (A), H2 (A)	dimeric	ancient
Red	172	H3 (A)	monomeric	ancient
Grey	172	H4 (A)	monomeric	ancient

*: The monomer types for blue and yellow layers were assigned previously [25],[26], and for the rest of the layers, in this paper. Division of AS into pure A type and AB type was introduced previously [1],[26].

**: Division of the old AS into “old” and “ancient” groups is introduced in this paper as a result of primate and cladistic analysis.

Figure 2 shows that same-color AS layers are shared by both arms of one chromosome, as well as by three different chromosomes. However, two solitary domains, grey (H4) and olive-green (H1H2), were observed. To find out if the counterparts of solitary domains were present elsewhere in the genome, we scanned the databases and found the arrays of sequences that mix well (Table S2 and Text S1) on chromosomes 1, 3, 4, 5 and 18 (grey) and 5 and 7 (olive-green). The yellow and blue layers corresponded to previously characterized SF4 (M1) and SF5 (R1R2), respectively [25],[26] (see Table 1 and Text S1). The genome-wide distribution of these families was documented previously [1], with additional examples provided in Table S6. The fact that same-color arrays from different chromosomes mix on phylogenetic trees (Figure 1C) confirms that, contrary to the new AS, the old AS had no chromosomal specificity and was homogenized genome-wide [1],[22] within a “suprachromosomal” layer.

The layers identified in Figure 2 showed no significant mixing with each other on phylogenetic trees (Figure 1). However, within some layers two or more closely related sub-domains could be discriminated (Figures S1 and S2). These sub-layers mixed with each other to some extent (Figure S3) and thus could not be formally identified as individual layers within the framework of this study. The nature and significance of this finer structure deserve further investigation (see Text S1 for details and discussion).

The layer pattern depicted in Figure 2 shows the structures partially symmetrical around the current centromere with most of the layers being shared between chromosomes. Same-color domains on different sides of the centromere mix well on phylogenetic trees (Figure 1B). This confirms that in many cases a new centromere arises in the midst of the old one, by amplification of a new AS variant, and moves the remnants of the old centromere sideways. However, emergence of evolutionary new centromeres [27],[28] and chromosomal rearrangements may predictably cause partial asymmetry. The same layers on different chromosomes combined with structural discontinuity in the succession of layers (e.g. monomeric – dimeric – monomeric) prove that the sequences seeding new centromeres were not picked up independently on each chromosome, but rather have spread by rounds of interchromosomal exchange and subsequent amplification, as was shown previously for the new AS families [1],[29],[30]. An alternative scenario of genome-wide homogenization with occasional exclusion of some domains or their parts is detailed in the “Discussion” section. Such a scenario would not support structural discontinuity and frequent symmetry and, therefore, cannot be solely responsible for the patterns described. However, it could explain the sub-layers noted above.

Interpretation of the layer structure proposed above allows a number of predictions. Hence, we moved on to verify it by phylogenetic, transposon distribution and divergence pattern analyses.

### The search for the last common human/primate AS layers

One would expect the extant primate taxa to share a certain number of ancestral layers with humans in a collinear succession up to the layer corresponding to the last common ancestor of humans and this particular primate. Ancestral layers may be followed by some primate-specific ones, corresponding to evolution of a primate branch after divergence from the human lineage. As this prediction is a particularly valuable tool in the hands of molecular anthropologists, we tested it by demonstrating the human lineage layers identified in this work in the genomes of various primate species. Samples of each layer were used to identify the most closely related sequences in the incomplete builds and collections of shotgun primate sequences in the GenBank. The AS monomers were extracted and aligned and phylogenetic trees constructed independently of human sequences. Consensus monomers of the branches were matched to consensus monomers of human layers and “mixing tests” were occasionally performed to verify the identity of the layers. As expected, each primate genome contained counterparts to some human lineage layers as well as the sequences specific for a particular primate branch. The results are summarized below and in Table 2, reference sequences are listed in Table S6.

**Table 2:** Search for human AS layers in various primates.

Primate	Grey H4 (PA7), 40 myr	Red H3 (PA5), 26 myr	Olive-green H1H2 (PA4/PA5), 23–26 myr	Yellow-striped V1 (PA4), 23 myr	Yellow M1 (PA3/PA4), 16–23 myr	Blue R1R2 (PA3), 16 myr	NEW
Tarsier (PA8), 58 myr							
*C.jacchus*, NWMs (PA7), 40 myr	+						
*M. mulatta*, OWMs (PA5), 25 myr	+	+	+				
Gibbon, apes (PA4), 18 myr	+	+	+	+	+		
Orangutan, great apes (PA3), 14 myr	+	+	+	+	+	+	
Gorilla, African apes (PA2), 7 myr	+	+	+	+	+	+	+

In the “Primate” column after the name of the primate and/or primate group, the youngest major L1 repeat shared with humans (in parentheses; our data) and the proposed age of the taxon [32] in million years (myr) are indicated. In other columns, the AS layer, the oldest L1 repeat found in the layer (in parentheses; our data), and the age of this L1 repeat estimated using mutation rate of 0.216% per myr [31] are indicated. In the new HOR arrays, L1 repeats are not present in significant numbers [2]. The presence of AS layers in certain species is indicated by “+”.

Notably, we failed to find any AS among abundant genomic sequences available for lemurs and tarsiers (not shown). Only grey layer (H4) sequences were found in the genome of a New World monkey (NWM) *Callithrix jacchus*, along with NWM-specific AS including S3S4 satellite [1]. The Old World monkeys (OWM) had grey (H4), red (H3) and olive-green (H1H2) layers plus a number of OWM-specific satellites including the one based on S1 and S2 types [1]. Therefore, we concluded that the grey layer belonged to an ancestor that pre-dated the NWM/OWM separation while the red and olive-green layers originated later, but were already present in a common ancestor of humans and OWM. Additionally, the yellow-striped (V1) and yellow (M1) layers, but no newer types, were found in gibbons. This indicated the existence of at least 2 entirely extinct taxa in the human lineage, the one with “red” (H3) centromeres between NWM and OWM, and the one with “yellow-striped” (V1) centromeres between OWM and gibbon ancestors. Recent analysis of HOR-like AS in gibbons [22] and our preliminary data (see Table S6 and Text S1) indicate the abundance of yellow-derived (M1) gibbon-specific AS sequences. In orangutans, the blue layer (R1R2) was present together with a number of the blue-derived orangutan-specific families composed of R1R2 dimers and longer HOR-like R1/R2 repeats (Table S6 and Text S1).

Contrary to expectations based on the old hybridization data [1], after an extensive search (over 11.4 Gb of WGS sequences screened), we failed to find new SFs in orangutans. Thus, the new AS is, in fact, specific to African apes, not great apes, as it was supposed previously. As expected, in gorilla and chimpanzee genomes, all the above layers plus the three new SFs 1, 2 and 3 were present (not shown, see Table S6 for reference sequences). As described above, certain types of AS sequences that were absent in some primate WGS reads were readily detectable in WGS collections of other primates. However, the conclusions based on the absence of findings should be treated with some degree of caution, as it is possible that the WGS reads were not comprehensive.

### L1 dating

To get another estimate of the age of AS layers identified in this work, we typed L1 retroposons integrated therein, as described previously [2]–[4]. The age of the oldest L1 elements found in an AS layer would indicate the time when it stopped homogenization and became available for insertions [2]. Table 2 (see also Table S3 and Text S1 for details) shows that the oldest L1 elements were identified as follows: PA3 in the blue layer; PA3 and just one PA4 in the yellow; PA4 in the yellow-striped; mostly PA4 and just two copies of PA5 in the olive-green layer. PA5 was the oldest L1 repeat in the red layer and PA7 in the grey (numerical number of L1 family increases with age [31]).

In order to relate these results to living primates' phylogeny we scored the L1 elements in the genomes of various primates looking for the elements active at the time of divergence of respective taxon with human lineage. In each genome the youngest major L1 repeat shared with humans was identified as follows: PB3 and PA15 (were active simultaneously [31]) for lemurs; PA8 for tarsiers; PA6 for NWM and PA5 for OWM. Gibbons had just a few PA3 and abundant PA4, orangutans had abundant PA3, gorillas and chimpanzees had abundant PA2 (Table 2 and Table S4).

Superimposing the above two sets of data, it can be concluded that the blue layer was already available for insertion shortly after orangutan divergence (PA3 was still active). The yellow layer, which was exposed to only a residual PA4 activity, if any, and a lot of PA3 activity, started to accumulate L1s between gibbon and orangutan divergence from the human lineage. The yellow-striped layer got its oldest L1s way after OWM divergence (PA4 is abundant) and the olive-green layer right before or right after that, as it still had PA5 activity. The red layer belonged to a more distant OWM - human ancestor (PA5 abundant) and the grey one to a common ancestor of OWM and NWM (PA7 present). The age of the layers may be roughly estimated as follows: new AS 7 myr, blue (R1R2) 14–16 myr, yellow (M1) 16–18 myr, yellow-striped (V1) 18–23 myr, olive-green (H1H2) 23–26 myr, red (H3) 26–40 myr, grey (H4) 40–58 myr.

As a temporary classification (see Table 1), we propose: (i) To term the AS forming centromeres of monkeys in human lineage “ancient AS” (types H1–H4; without suprachromosomal family names), (ii) to keep the term “old AS” only for lower ape layers, namely V1 (yellow-striped; SF6), M1 (yellow; SF4) and R1R2 (blue; SF5) and, (iv) to apply the term “new AS” to African ape-specific SFs 1, 2, and 3 [1] (see Text S1 for details).

### Divergence analysis in AS domains

It is expected that the closer the layer is to a current centromere the younger it is and the less is the divergence between monomers (or dimers) within the array. Table 3 shows that in all cases the pattern of divergence does not contradict this prediction. Divergence figures for same-color domains on one chromosome and on different chromosomes are in remarkable concordance.

**Table 3:** Average identity of monomers in AS layers.

Layer/chromosome	Grey H4	Red H3	Olive H1	Green H2	Yellow-striped V1	Yellow M1
Xp	0.711	0.733	0.785	0.790	0.818	0.845
	0.043	0.046	0.038	0.038	0.033	0.038
	292	194	301	282	288	28
Xq		0.737			0.812	0.833
		0.040			0.038	0.024
		468			85	88
						0.837
						0.023
						136
8p		0.732				
		0.039				
		1566*				
8q		0.725				0.812
		0.040				0.035
		1238*				76
17p		0.730			0.803	
		0.042			0.039	
		168			101	
		0.723				
		0.045				
		143				
17q		0.723				0.850
		0.045				0.046
		167				23
Mean within a monomer type (%)	71	73	79	79	81	84
Mean between monomer types (%)	66	67	70	70	72	69

The mean identity of monomers in pairwise comparisons within each array (upper line), standard deviation (middle line), and the number of monomers are indicated for each AS layer, as depicted in Figure 2. In cases where two domains of the same color are present on one chromosome arm, figures for both of them are presented separately; multiple red arrays in 8q are presented summarily. In cases marked by an asterisk, only 1,000 monomers were used in comparisons. In the two bottom lines, the mean of each column representing the identity across a whole layer and the mean identity obtained in “between layers” comparisons (this layer to all the others) are presented. It can be seen that the intra-array divergence and hence the age of the arrays decrease towards the centromere.

We next tested if the divergence in each layer would match its proposed age. Orthologous comparisons of the grey (H4) domains from a number of primate species [4],[24] (Table S5) yield a “normal” mutation rate of about 0.2% per million years (0.17%–0.21%; see Text S1), similar to surrounding euchromatin [4],[24] and L1 repeats [31]. However, the age of the layers calculated from intra-array divergence figures, using this rate, clearly contradicts our primate and L1 dating (Table 4). This “calculated age” is about twice as old and out of line with all accepted taxon age estimates [32]. Conversely, mutation rate calculated using intra-array divergence and the age of layers estimated from primate and L1 dating, ranges 0.4%–0.6% and is 2 to 3 times higher than normal. To explain this discrepancy we propose that a period of hypermutability occurs in homogeneous AS array after the centromere moves away and homogenization stops. The high mutation rate may somehow be caused or conditioned by near perfect identity of centromeric repeats and gradually subsides upon accumulation of mutations and repeat divergence. The normal rate applies only to the “long dead” arrays. The high rate applies to the “hypermutability” stage (“freshly abandoned” centromeres) and possibly to the “homogeneous” stage of array evolution (active centromeres). In the latter case, it may drive the rapid concerted evolution of homogenous HOR domains [24]. Hypermutability may be caused by low fidelity DNA synthesis, if secondary structures in AT-rich satellite or high density of replication origins [33] cause fork stalling and trigger repair mechanisms using error-prone DNA polymerases [34] (see Text S1 for more details).

**Table 4:** Age of AS layers calculated by different methods.

Layer	“Calculated age” by array divergence (myr)	Age by primate & L1 dating (myr)
Grey (H4)	92	40–58
Red (H3)	84	26–40
Olive-green (H1H2)	62	23–26
Yellow-striped (V1)	55	18–23
Yellow (M1)	45	16–18

The “mock” age estimates calculated from intra-array divergence on the basis of 0.2% mutation rate are compared to “valid” estimates obtained from L1 and primate dating. The age calculated from the divergence figures is about twice as old as provided by other estimates.

## Discussion

### The origin of species is written in centromeres

The record of human evolutionary lineage revealed in the centromeres appears to be relatively consistent and easy to interpret. There is remarkable concordance between primate studies, L1 dating and divergence grading. However, our conclusions have to be verified further upon availability of more comprehensive primate sequencing data. Notably, living primates faithfully represent the succession of major taxa in the human lineage. The only two entirely extinct taxa are represented by red (H3) and yellow-striped (V1) layers. The controversial fossil record [32],[35] offers at least three candidate extinct families: Propliopithecidae (Catopithecus and Aegyptopithecus; 33–35 myr), Pliopithecidae (Proconsul; 17–27 myr) and Dryopithecidae (Dryopithecus; 9–14 myr). The first two match the red (26–40 myr) and yellow-striped (18–23 myr) layers pretty well and the third is not supported by a separate AS layer, which may mean it is a sister clade of either African apes or Pongidae (orangutan family). The exact positions of extinct taxa in human lineage are hotly debated [35] and comprehensive analysis of AS layers in extant primates and man would help to resolve this problem. The possibility to number and locate the extinct ancestral taxa on the evolutionary tree and to distinguish the ancestor from the descendant even in two-species comparisons is unique to the AS record. As there is every reason to believe that centromeric layers are not limited to AS and primates, the method has vast potential for phylogenetic studies.

### Mechanistic scenarios of AS evolution

Figure 2 displays imperfect symmetry of AS layers around the current centromere. One can potentially explain it in two different ways. The process creating layers can be asymmetrical in nature, and the elements of symmetry may appear randomly as a matter of coincidence. Alternatively, the process may be intrinsically symmetrical, but the symmetry is imperfect for a number of random historical reasons like formation of evolutionary new centromeres, chromosomal rearrangements, etc.

A possible scenario for the asymmetrical process would be continuous genome-wide homogenization and concerted evolution of active centromeres, identical on all chromosomes. From time to time for various reasons (inversion, insertion of a long mobile element, amplification of another tandem repeat, etc.) parts of individual arrays get cut off the bulk of the array and hence are excluded from homogenization (a “segment cut off scenario”). As KARM presumably brings efficient homogenization only to the current centromere, and only one centromere per chromosome can be maintained, the cut off part looses both homogenization and centromeric function and becomes a dead segment. An epigenetic mark [20] is likely to be involved in the stable choosing of only one of the segments as a centromere, in this scenario. If two segments on both arms of one chromosome die at about the same time, they would form a symmetrical structure. Dead segments in different chromosomes, excluded from homogenization at the same time, would form a “same-color” suprachromosomal layer. However, such coincidences and hence the elements of symmetry should be rare. Also, structurally discontinuous patterns like a succession of monomeric – dimeric – monomeric layers are hardly possible. Therefore, this process cannot be solely responsible for centromeric evolution.

A mechanism for the symmetrical process was described as the only one possible for the new chromosome-specific SFs [1],[29],[30], which are represented by structurally different HORs on each chromosome. It includes a series of interchromosomal transfers and amplification events, facilitated, as we propose, by KARM, which is also responsible for homogenization (“interchromosomal transfer/amplification scenario”). In most cases, new variants come from another location, insert into the active centromere, split and inactivate it by luring the kinetochore to the new array and move the remains sideways as a result of self-expansion. Potentially, this process could be solely responsible for the layer pattern revealed in chromosomes 8, 17 and X. However, depending on the extent of symmetry and interchromosomal similarity of the layer patterns in the rest of the genome, some combination of the two scenarios may appear to be parsimonious. Notably, in this work we studied only the centromeres with SF2 (chromosome 8) and SF3 (chromosomes 17 and X) HOR domains. However, our unpublished preliminary results show that SF1 centromeres are flanked by the same types of old and ancient AS sequences (see chromosome 7 sequences in Tables S3 and S6).

Each new expansion of an AS variant covered many chromosomes and occurred in a relatively short time, as the order of layers is more or less conserved between chromosomes. Obviously, an expanding variant had to possess some sequence novelty, which attracted the kinetochore [1],[36]. For instance, a new or better fitting protein-binding site might make a satellite repeat a more attractive centromere, as may be exemplified by evolutionarily recent recruitment of CENP-B protein to primate centromeres [26],[37],[38]. Initially new sequence variants may arise in poorly homogenized areas such as dead segments, borders of current centromeric arrays, etc. A successful variant has to accidentally insert into a current centromere, win over the kinetochore and self-expand. Apparently, once such “better” centromeric sequence appeares on one chromosome, it has a good chance to invade other chromosomes very quickly. Together with the tendency of a new variant to integrate/amplify in the current centromere, not in the old layers, it suggests that KARM may take part in interchromosomal transfer and/or integration and amplification in new locations. When a neocentromere is formed on unique DNA, KARM may be used to seed and amplify centromeric repeats at the new site. It is also likely that only a sequence integrated in the current centromere may easily acquire the centromeric epigenetic mark [20]. Additional details of specific scenarios are provided in Text S1.

### Centromere plasticity

The centromere is remarkable for its plasticity. Centromeric DNA and proteins are subject to phylogenetic variation very much unlike other components of chromatin and cell division machinery [21]. Here we show that constant generation of new AS variants and perhaps their competition for centromeric function resulted in serial waves of AS expansion in the course of primate evolution. Each wave led to emergence of new underlying sequences in active centromeres of many chromosomes. It was demonstrated previously that in the genomes of monkeys, A-type AS, as a rule, is the same in all chromosomes and hence is homogenized throughout the whole genome [1],[22]. On the contrary, the new AB-type AS which is present in the genomes of African apes is chromosome-specific and, as a rule, is effectively homogenized only within one chromosome. According to our model, an AS layer unites the arrays which (i) have a common origin, (ii) were active centromeres at the same time, and (iii) at that time were homogenized throughout the genome as a single entity. Points i and ii are also valid for the new SFs, but point 3 is not, otherwise SFs and AS layers are the same. This difference reflects a shift from genome-wide to chromosome-specific homogenization.

Centromeric function *per se* can be performed by unique sequences as exemplified by neocentromeres [27],[39] and the centromeres of budding yeast [40]. However, natural centromeres of all higher organisms are made of highly repeated sequences, hinting perhaps at some additional function. Two such functions, not mutually exclusive and perhaps even interrelated, have been discussed. Cohesion of centromeres, tension sensing and signalling to the spindle assembly checkpoint, may be provided by the formation and resolution of PICH threads [8]–[12]. If PICH threads are formed by recombination intermediates, they may just show the proposed kinetochore-associated recombination machine (KARM) at work. Vast satellite arrays provide phenotypically silent DNA to form the threads, which, therefore, are indifferent to breakage, occasional erroneous repair, etc. On the other hand, the features of primate highly repeated centromeres, such as (i) tandem structure prone to recombination, (ii) putative possession of its own recombination machine, (iii) presence of a divergent dead zone that provides a good source of new sequence variants, and finally (iv) alleged propensity to go through hypermutability periods, seem to constitute a special “plasticity” adaptation evolved to ensure that from time to time a new centromere would arise in a stochastic manner. The concept of centromeric speciation [21] speculates on possible evolutionary benefit of such an adaptation. It suggests that centromere plasticity may play a role in generation of new species by providing partial reproductive isolation of a karyological variant with a new centromeric layer. Establishment and separation of incipient species may proceed via mechanisms described for other types of chromosomal speciation [41].

## Materials and Methods

The NCBI website (www.ncbi.nlm.nih.gov/) was used to extract human centromeric regions and the BLAST server (www.ncbi.nlm.nih.gov/blast/Blast.cgi) to search for AS-containing human and primate clones and WGS reads. AS monomers were identified by PERCON similarity search [2], extracted and classed into monomeric types and SFs by a Bayesian classifier [2] and aligned by CLUSTALW [42]. After manual inspection, a small number of evidently abnormal monomers were discarded. Phylogenetic trees were constructed using the PHYLIP 3.65 package (http://evolution.genetics.washington.edu/phylip.html). DNA distance matrix was calculated using the F84 method and trees were constructed by UPGMA and neighbor-joining methods. Similar neighbor-joining trees were obtained with the MEGA4 package (www.megasoftware.net) using the same monomer sets, and most major branches were verified by an interior branch test (>90%) in 500 replicates as described [4]. To identify a group of monomers as distinct AS domain we used four main steps: (i) The “branching test” shows that monomers are on the same branch of the monomer phylogenetic tree and hence are closely related and have presumably arisen by amplification of a single ancestral sequence; (ii) the “compact residence test” verifies that all these closely related monomers are concentrated in separate array(s) without significant interspersion with monomers from other branches; (iii) the “structural test” evaluates the divergence in the group, reconstructs the ancestral sequence by derivation of an appropriate group consensus and places it in AS classification by establishing the relationships with other AS monomeric types. (iv) Finally the “mixing test” shows that the monomers from differently located domains mix on one branch of a monomer tree and hence have arisen from a single ancestor and were once homogenized as one entity (i.e. belong to one AS “layer”). Layer-specific AS sequences, in human and primate genomes, were searched by using samples of layers as queries in a BLAST search of human or primate NCBI databases, including WGS assemblies and trace collections of shot-gun sequences. WGS reads containing AS were used as such without any experimental verification of their centromeric location.

Non-AS repeats were identified by RepeatMasker (www.RepeatMasker.org). The same program was used for L1 classification.

Mutation rates were calculated using Jukes and Cantor formula [43] (see Text S1).

## Supporting Information

Figure S1 Schematic representation of Xp (A) and Xq (B) pericentromeric regions. A colored map is presented in the bottom part. The colors of AS arrays (thick line; changes of directionality are indicated) correspond to the ones in Figures 1 and 2. In addition, the orange (Xp) and lilac (Xq) subdomains within the red layer and bright green (Xq) subdomain within the yellow-striped layer are depicted in respective colors (see Text S1 and Figure S2). Interspersed repeated elements are shown as light blue raised boxes with directionality indicated by the slope. Other satellites are indicated similarly by thick bands. Above the map, the mean similarity to 12-mer DXZ1 HOR (diamonds) and relative similarity to the ALPHA-ALL [26] consensus monomer (triangles) are plotted. In the *HOR similarity plot*, each dot corresponds to a monomer and shows mean similarity of 12-mer starting with this monomer to DXZ1 HOR. There are gaps around inversion breakpoints and insertions, because a continuous unidirectional stretch of 12 AS monomers to the right of a given monomer is needed to calculate each point in this plot. In HOR arrays (light blue thick line), the pattern is periodic, and three levels of similarity corresponding to three types of alignment can be seen: between 95% and 100% for “in register HOR” alignment, between 75% and 80% for alignment of pentameric ancestral repeats within the HOR, and between 70% and 75% for completely non-register alignment. In non-HOR regions, similarity ranges from a little less than 60% to a little over 75%, and each layer has a characteristic range. Transition between HOR and monomeric region is abrupt with only 1–2 divergent HORs on the border. In the *ALPHA-ALL similarity plot*, relative similarity (rs) to ALPHA-ALL is shown for each monomer as well as a smoothing curve (over 4 monomers). Rs is calculated as alignment score divided by reward for a match multiplied by the length of alignment, where alignment score is a number of matches multiplied by reward for a match minus number of gaps multiplied by gap opening penalty minus a number of nucleotides in gaps multiplied by a penalty for gap extension minus a number of mismatches multiplied by a penalty for mismatch (i.e. affine scoring scheme was used). A periodic pattern is seen in HOR arrays, and every dead layer is characterized by its own level of similarity. The most notable is the transition between old and ancient AS (olive-green/yellow-striped border in Xp and yellow-striped/red in Xq). Rs>0.6 defines old arrays and rs<0.6 defines ancient arrays.
(0.36 MB TIF)

Figure S2 Alpha satellite sublayers in human chromosomes 8, 17, and X. Same as Figure 2, but orange (Xp and 8q) and lilac (Xq and 17) sublayers within the red layer and bright green subdomain within the yellow-striped layer (Xq) are depicted in respective colors, as described in Text S1.
(0.29 MB TIF)

Figure S3 Sublayers in the red and yellow-striped layers from chromosomes 8, 17, and X. Same phylogenetic tree as in Figure 1C, but orange and lilac sublayers within the red layer and bright green (Xq) subdomain within the yellow-striped domain on Xq are depicted in respective colors, as described in Text S1 and shown in Figure S2. The yellow monomers are shown in yellow and the yellow-striped monomers are shown in brass color.
(1.35 MB TIF)

Table S1 Position of AS regions on chromosomes X, 8, and 17 with respect to genomic contigs and build 36.2. “Start” is a start of a contig or clone in build 36.2. “Length” is the length of a contig or clone.
(0.05 MB DOC)

Table S2 X chromosome AS layers are found in multiple locations on human chromosomes. For clones from chromosomes 17 and X, see Table S1. Where other layers are present, the regions referred to are indicated in parenthesis. *See also additional sequences in Table S6.
(0.03 MB DOC)

Table S3 Statistics of human L1 types in various AS layers. The upper part of the table presents the data obtained for sequences listed in Tables S1 and S2. For the layers underrepresented in this sample, “ADDITIONAL” sequences were identified and scored (human sequences listed in Table S6). The total length of the sequences scored in each layer is indicated in the “Length” column in kb. In other columns the figures represent the number of L1 repeats scored in each layer. The figures for the oldest major L1 family present in respective AS layer are marked in boldface and for the minor oldest family are underlined.
(0.06 MB DOC)

Table S4 Statistics of human L1 types in various primate genomes. *PA1 or L1Hs is a human-specific L1 species. ** There is no PA9 family, but PA8 and PA8A instead, which were treated collectively for the purposes of this analysis, because they were active simultaneously and their combined copy number about equals that of other families; the copy number of PA2, 6, and 10 is about twice as little [31]. Bold and underline: The figures for the youngest major family which was active in the genome of respective primate species are marked in boldface and for the minor youngest family are underlined. 1–3 copies were considered as possible classification mistakes or rare recombination events.
(0.03 MB DOC)

Table S5 Similarity between 3 kb of the most distal part of the grey domain in various primates.
(0.02 MB DOC)

Table S6 Human sequences used in “additional” L1 scoring in Table S3 and reference sequences for primate AS layers.
(0.06 MB DOC)

Text S1 Supplemental information on details of AS analysis, its interpretation, and how it relates to previous data.
(0.09 MB DOC)
