# Supplementary figures and images for: The Evolutionary Origin of Man Can Be Traced in the Layers of Defunct Ancestral Alpha Satellites Flanking the Active Centromeres of Human Chromosomes
Source: PLoS Genet. 2009 Sep 11;5(9):e1000641. doi: 10.1371/journal.pgen.1000641 (PMC2729386; doi:10.1371/journal.pgen.1000641)

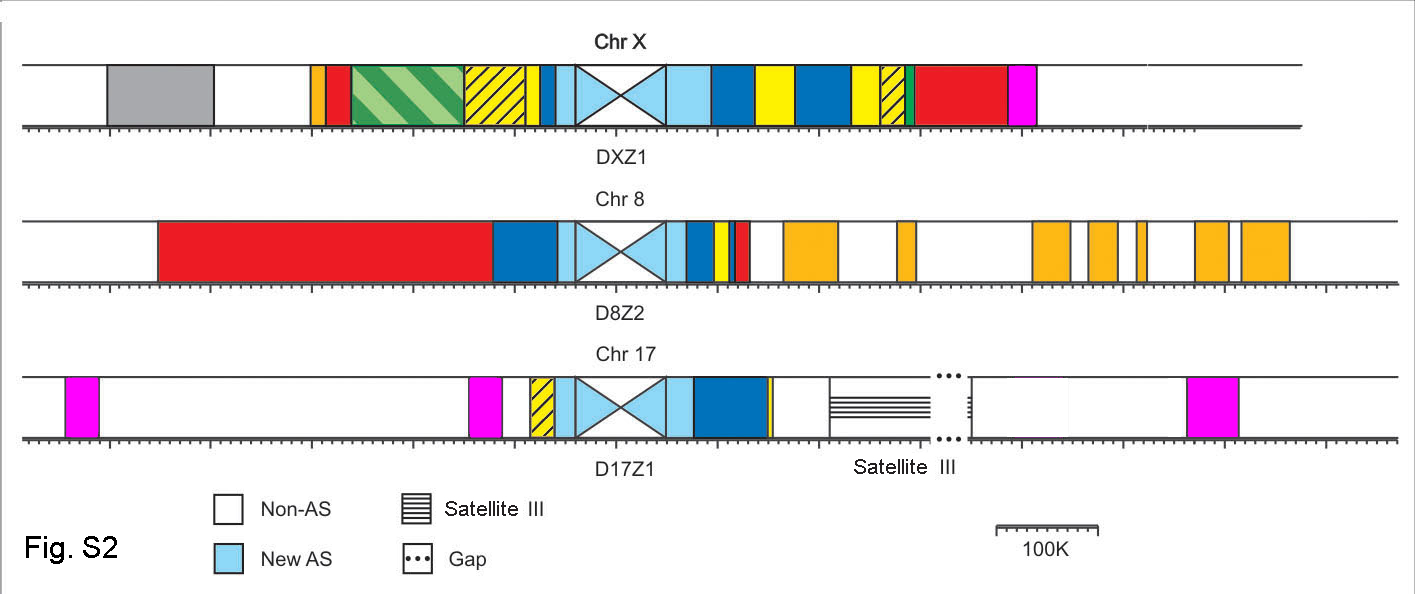

Supplement: Figure S2 — Alpha satellite sublayers in human chromosomes 8, 17, and X. Same as Figure 2, but orange (Xp and 8q) and lilac (Xq and 17) sublayers within the red layer and bright green subdomain within the yellow-striped layer (Xq) are depicted in respective colors, as described in Text S1. (0.29 MB TIF) [file pgen.1000641.s002.tif]

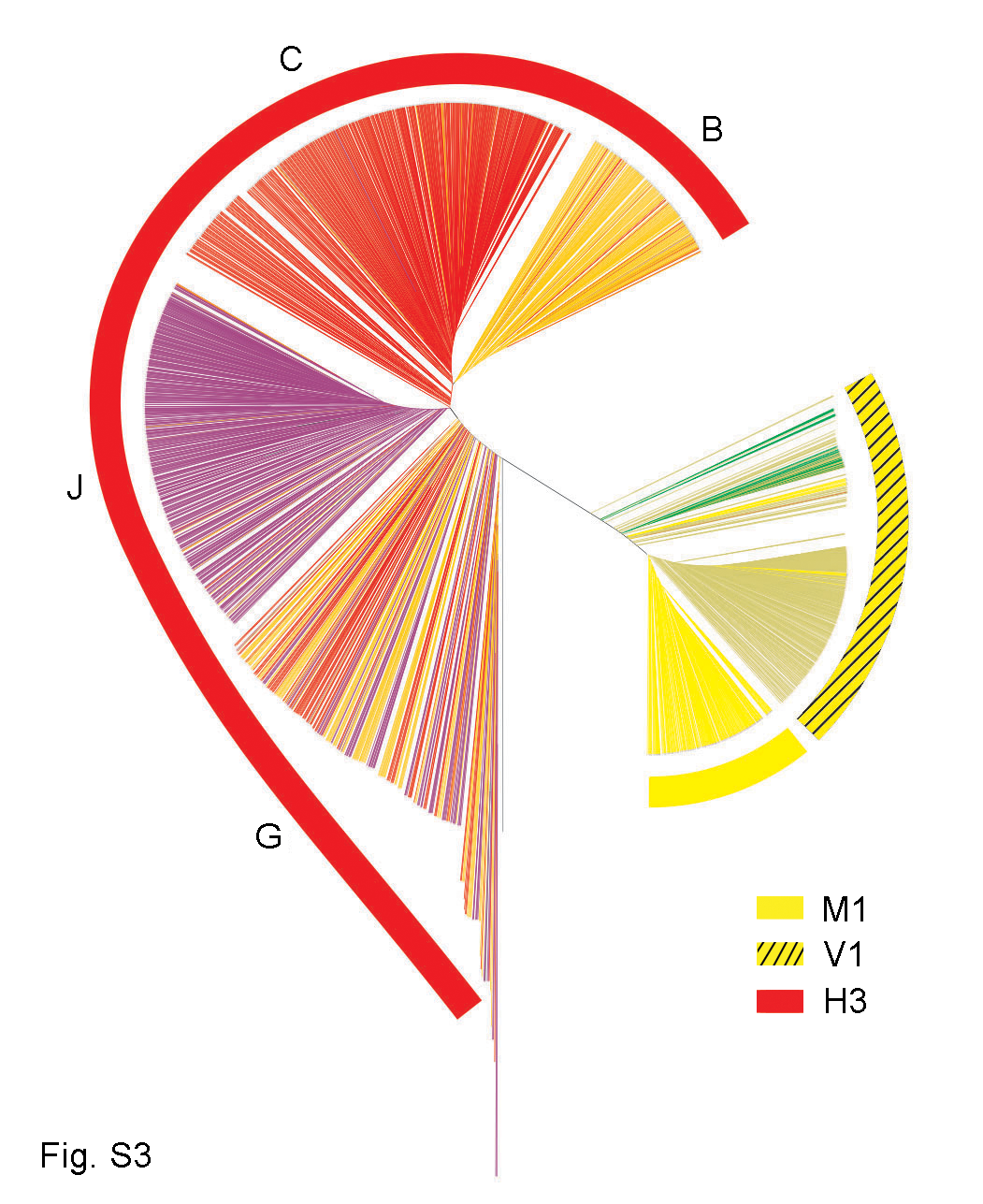

Supplement: Figure S3 — Sublayers in the red and yellow-striped layers from chromosomes 8, 17, and X. Same phylogenetic tree as in Figure 1C, but orange and lilac sublayers within the red layer and bright green (Xq) subdomain within the yellow-striped domain on Xq are depicted in respective colors, as described in Text S1 and shown in Figure S2. The yellow monomers are shown in yellow and the yellow-striped monomers are shown in brass color. (1.35 MB TIF) [file pgen.1000641.s003.tif]
