# Supplementary material for: The Evolutionary Origin of Man Can Be Traced in the Layers of Defunct Ancestral Alpha Satellites Flanking the Active Centromeres of Human Chromosomes
Source: PLoS Genet. 2009 Sep 11;5(9):e1000641. doi: 10.1371/journal.pgen.1000641 (PMC2729386; doi:10.1371/journal.pgen.1000641)
Supplement: Table S1 — Position of AS regions on chromosomes X, 8, and 17 with respect to genomic contigs and build 36.2. “Start” is a start of a contig or clone in build 36.2. “Length” is the length of a contig or clone. (0.05 MB DOC) [file pgen.1000641.s004.doc]

**Table S1.** **Position of AS regions on chromosomes X, 8 and 17 with respect to genomic contigs and build 36.2.**

| **Chromosome** | **In contig or BAC** | **Strand** | **In build 36.2** |
| --- | --- | --- | --- |
| **ChrXp** | **NT_011630.14** Start=52462640 Length=6136098 | | |
| First monomer start | 5642534 | C | 58105173 |
| Last monomer end | 6136097 | C | 58598736 |
| **ChrXq** | **NT_011669.15** Start=61598738 Length=14971611 | | |
| First monomer start | 3 | C | 61598740 |
| Last monomer end | 363871 | C | 61962608 |
| **Chr8p** | **NT_007995.14** Start=29798770 Length=14159284 | | |
| First monomer start | 13747766 | + | 43546535 |
| Last monomer end | 14159273 | + | 43958042 |
| **Chr8q** | **NT_023678.15** Start=49958053 Length=1291149 | | |
| First monomer start | 3 | + | 49958055 |
| Last monomer end | 617484 | + | 50575536 |
| **Chr17p** | **NT_024862.13** Start=21607210 Length=579924 | | |
| First monomer start | 75628 | C | 21682837 |
| Last monomer end | 579924 | + | 22187133 |
| **Chr17q** | **NT_010799.14** Start=22287134 Length=9412828 | | |
| First monomer start | 212855 | + | 22499988 |
| Last monomer end | 261433 | + | 22548566 |
| **Chr17q** | **AC141299.1** (HTGS) Not placed. Length=164356 | | |
| First monomer start | 12049 | + | Not placed |
| Last monomer end | 163352 | C | Not placed |
| **Chr17q** | **AC083828.3** (HTGS) | Not placed | Length=194734 |
| **Chr17q** | **NW_927772.1** (Celera) | Not placed | Length=254856 |

“Start” is a start of a contig or clone in build 36.2. “Length” is the length of a contig or clone.
