# Supplementary material for: The Evolutionary Origin of Man Can Be Traced in the Layers of Defunct Ancestral Alpha Satellites Flanking the Active Centromeres of Human Chromosomes
Source: PLoS Genet. 2009 Sep 11;5(9):e1000641. doi: 10.1371/journal.pgen.1000641 (PMC2729386; doi:10.1371/journal.pgen.1000641)
Supplement: Table S2 — X chromosome AS layers are found in multiple locations on human chromosomes. For clones from chromosomes 17 and X, see Table S1. Where other layers are present, the regions referred to are indicated in parenthesis. *See also additional sequences in Table S6. (0.03 MB DOC) [file pgen.1000641.s005.doc]

**Table S2. X chromosome AS layers are found in multiple locations on human chromosomes.**

| Layer | Chromosomes | Clones |
| --- | --- | --- |
| Grey | 1,3,4,5,18,X, | Chr1 AC113173  Chr3 AC024967 AC129677  Chr4 AC108018  Chr5 AC108093 AC024565  Chr18 AC026971 AC068204 |
| Olive/Green | 5,7,X | Chr5 AC122694 (68-110Kb)  Chr7q Nt_007758 (165-484Kb) |
| Yellow-  Striped* | 7,17,X | Chr7q Nt_007758 (67-102Kb) |

For clones from chromosomes 17 and X see Table S1. Where other layers are present, the regions referred to are indicated in parenthesis.

* See also additional sequences in Table S6.
