# Supplementary material for: The Evolutionary Origin of Man Can Be Traced in the Layers of Defunct Ancestral Alpha Satellites Flanking the Active Centromeres of Human Chromosomes
Source: PLoS Genet. 2009 Sep 11;5(9):e1000641. doi: 10.1371/journal.pgen.1000641 (PMC2729386; doi:10.1371/journal.pgen.1000641)
Supplement: Table S3 — Statistics of human L1 types in various AS layers. The upper part of the table presents the data obtained for sequences listed in Tables S1 and S2. For the layers underrepresented in this sample, “ADDITIONAL” sequences were identified and scored (human sequences listed in Table S6). The total length of the sequences scored in each layer is indicated in the “Length” column in kb. In other columns the figures represent the number of L1 repeats scored in each layer. The figures for the oldest major L1 family present in respective AS layer are marked in boldface and for the minor oldest family are underlined. (0.06 MB DOC) [file pgen.1000641.s006.doc]

**Table S3.** **Statistics of human L1 types in various AS layers.**

| Layer | Length | PA1 | PA2 | PA3 | PA4 | PA5 | PA6 | PA7 | PA8 | PA10 |
| --- | --- | --- | --- | --- | --- | --- | --- | --- | --- | --- |
| Grey | 148 | 0 | 0 | 2 | 0 | 1 | 0 | **4** | 0 | 0 |
| Red | 868 | 0 | 2 | 3 | 7 | **4** | 0 | 0 | 0 | 0 |
| Olive/green | 494 | 1 | 5 | 5 | **7** | 2 | 0 | 0 | 0 | 0 |
| Yellow-striped | 90 | 0 | 2 | 2 | **2** | 0 | 0 | 0 | 0 | 0 |
| Yellow | 87 | 0 | 2 | **4** | 0 | 0 | 0 | 0 | 0 | 0 |
| Blue | 218 | 1 | 5 | **3** | 0 | 0 | 0 | 0 | 0 | 0 |
| ADDITIONAL* |  |  |  |  |  |  |  |  |  |  |
| Yellow-striped | 408 | 3 | 5 | 7 | **10** | 0 | 0 | 0 | 0 | 0 |
| Yellow | 355 | 1 | 2 | **18** | 1 | 0 | 0 | 0 | 0 | 0 |
| Blue | 832 | 5 | 13 | **15** | 0 | 0 | 0 | 0 | 0 | 0 |
| TOTAL |  |  |  |  |  |  |  |  |  |  |
| Grey | 148 | 0 | 0 | 2 | 0 | 1 | 0 | **4** | 0 | 0 |
| Red | 868 | 0 | 2 | 3 | 7 | **4** | 0 | 0 | 0 | 0 |
| Olive/green | 494 | 1 | 5 | 5 | **7** | 2 | 0 | 0 | 0 | 0 |
| Yellow-striped | 498 | 3 | 7 | 9 | **12** | 0 | 0 | 0 | 0 | 0 |
| Yellow | 442 | 1 | 4 | **22** | 1 | 0 | 0 | 0 | 0 | 0 |
| Blue | 1050 | 6 | 18 | **18** | 0 | 0 | 0 | 0 | 0 | 0 |

The upper part of the table presents the data obtained for sequences listed in Tables S1 and S2. For the layers underrepresented in this sample, “ADDITIONAL” sequences were identified and scored (human sequences listed in Table S6). The total length of the sequences scored in each layer is indicated in the “Length” column in kb. In other columns the figures represent the number of L1 repeats scored in each layer. The figures for the oldest major L1 family present in respective AS layer are marked in boldface and for the minor oldest family are underlined.
