# Supplementary material for: The Evolutionary Origin of Man Can Be Traced in the Layers of Defunct Ancestral Alpha Satellites Flanking the Active Centromeres of Human Chromosomes
Source: PLoS Genet. 2009 Sep 11;5(9):e1000641. doi: 10.1371/journal.pgen.1000641 (PMC2729386; doi:10.1371/journal.pgen.1000641)
Supplement: Table S4 — Statistics of human L1 types in various primate genomes. *PA1 or L1Hs is a human-specific L1 species. ** There is no PA9 family, but PA8 and PA8A instead, which were treated collectively for the purposes of this analysis, because they were active simultaneously and their combined copy number about equals that of other families; the copy number of PA2, 6, and 10 is about twice as little [31]. Bold and underline: The figures for the youngest major family which was active in the genome of respective primate species are marked in boldface and for the minor youngest family are underlined. 1–3 copies were considered as possible classification mistakes or rare recombination events. (0.03 MB DOC) [file pgen.1000641.s007.doc]

**Table S4. Statistics of human L1 types in various primate genomes.**

| Primate | PA1  (Hs)* | PA2 | PA3 | PA4 | PA5 | PA6 | PA7 | PA8 +  PA8A ** | PA10 |
| --- | --- | --- | --- | --- | --- | --- | --- | --- | --- |
| Tarsier | 0 | 1 | 2 | 0 | 0 | 1 | 4 | **15** | 6 |
| *C.jacchus*  NWM | 1 | 0 | 1 | 0 | 0 | 2 | **135** | 39 | 12 |
| *M. mulatta*  OWM | 0 | 0 | 1 | 0 | **42** | 3 | 14 | 9 | 4 |
| Gibbon  apes | 0 | 0 | 5 | **46** | 5 | 5 | 14 | 3 | 9 |
| Orangutan  great apes | 0 | 1 | **53** | 20 | 12 | 6 | 23 | 31 | 14 |
| Gorilla  African apes | 1 | **11** | 8 | 11 | 8 | 2 | 17 | 9 | 11 |

* PA1 or L1Hs is a human-specific L1 species.

** There is no PA9 family, but PA8 and PA8A instead, which were treated collectively for the purposes of this analysis, because they were active simultaneously and their combined copy number about equals that of other families; the copy number of PA2, 6, and 10 is about twice as little [31].

The figures for the youngest major family which was active in the genome of respective primate species are marked in boldface and for the minor youngest family are underlined. 1-3 copies were considered as possible classification mistakes or rare recombination events.
