# Supplementary material for: The Evolutionary Origin of Man Can Be Traced in the Layers of Defunct Ancestral Alpha Satellites Flanking the Active Centromeres of Human Chromosomes
Source: PLoS Genet. 2009 Sep 11;5(9):e1000641. doi: 10.1371/journal.pgen.1000641 (PMC2729386; doi:10.1371/journal.pgen.1000641)
Supplement: Table S5 — Similarity between 3 kb of the most distal part of the grey domain in various primates. (0.02 MB DOC) [file pgen.1000641.s008.doc]

**Table S5. Similarity between 3kb of the most distal part of the grey domain in various primates.**

Chimpanzee Gorilla Orangutan *Macaca Papio Cercopithecus*

*mulatta anubis aethiops*

AC140661 AC147693 AC147722 AC147688 AC147591 AC147690

-----------------------------------------------------------------------------

Man 98.7% 97.6% 94.8% 90.4% 91.5% 91.1%

Chimpanzee 98.0% 95.0% 90.6% 90.9% 91.0%

Gorilla 94.7% 90.3% 90.7% 90.4%

Orangutan 90.1% 90.8% 90.3%

*M. mulatta* 98.4% 97.3%

*P. anubis* 97.6%

Tree produced by nearest neighbor joining procedure

100% 99% 98% 97% 96% 95% 94% 93% 92% 91% 90%

!----!----!----!----!----!----!----!----!----!----!----

Man o------+--+--------------+----------------+-------------

Chimpanzee o------! ! ! !

Gorilla o---------! ! !

Orangutan o------------------------! !

*M.mulatta* o-------+---+-----------------------------!

*P.anubis* o-------! !

*C.aethiops* o-----------!
