# Supplementary material for: The Evolutionary Origin of Man Can Be Traced in the Layers of Defunct Ancestral Alpha Satellites Flanking the Active Centromeres of Human Chromosomes
Source: PLoS Genet. 2009 Sep 11;5(9):e1000641. doi: 10.1371/journal.pgen.1000641 (PMC2729386; doi:10.1371/journal.pgen.1000641)
Supplement: Table S6 — Human sequences used in “additional” L1 scoring in Table S3 and reference sequences for primate AS layers. (0.06 MB DOC) [file pgen.1000641.s009.doc]

**Table S6. Human sequences used in “additional” L1 scoring in Table S3 and reference sequences for primate AS layers.**

| Primate | Clone accession number and chromosome, if known | Layers and comments |
| --- | --- | --- |
| Human | AC069350 chromosome 3 | Blue/yellow |
|  | AC068825 chromosome 3 | Blue |
|  | AC129664 chromosome 4 | Blue/yellow |
|  | AC127385 chromosome 4 | Blue/yellow |
|  | AC020949 chromosome 5 | Blue |
|  | AC125787 chromosome 5 | Blue/yellow/ancient |
|  | AC019063 chromosome 7 | SF1/blue/yellow/yellow-striped /ancient |
|  | Nt_038037 chromosome 19 | Blue |
|  | AC136499 chromosome 19 | Blue |
|  | AL358116 chromosome 20 | Blue |
|  | AC128676 | Yellow-striped/ancient |
|  | AC144523 | Blue/yellow/yellow-striped/ancient |
| Gorilla *G. gorilla* | ti=757619844  757620019, 757619956 | SF1 |
|  | ti=757619906, 757620380,  757621988 | SF2 |
|  | X56287 | SF3, X-specific |
|  | AC147693, AC198821,  AC202239, AC199985 | BACs form Xp contig  Grey/red/olive-green/yellow-striped/yellow/blue |
| Orangutan  *P. pygmaeus* | AC147722 | grey |
|  | AC204792,AC207089 | Red /olive-green/yellow-striped |
|  | ABGA01055294 | Yellow/blue |
|  | ti=885841334 | Blue, HOR-like, homogeneous, high copy number |
| Gibbon  *N. leucogenys* | ti=1236414561, 1249311757,  1259048821 | grey |
|  | CT954299 (47K-77K) | Red |
|  | ti=1255332931, 1259057267,  1259060447 | Red |
|  | ti=1236427070, 1236571618,  1236573496 | Olive-green |
|  | ti=1237748956, 1252827322,  1252827756 | Yellow-striped |
|  | ti=1242596243, 1252839975,  1254962914 | Yellow |
|  | ti=2054969403 | SF4, yellow, major HOR-like AS, gibbon-specific |
| *M. mulatta* | NW_001218125 | Grey/red/olive-green, monkey-specific |
|  | AANU01000162 | S1S2 monkey-specific homogeneous satellite |
| *C. jacchus* | ti=1052388340, 1017768879,  1025086157 | grey |
|  | ti=1000238351,1000238514,  1000239684 | S3S4 monkey-specific homogeneous satellite |
