# Supplementary material for: The Evolutionary Origin of Man Can Be Traced in the Layers of Defunct Ancestral Alpha Satellites Flanking the Active Centromeres of Human Chromosomes
Source: PLoS Genet. 2009 Sep 11;5(9):e1000641. doi: 10.1371/journal.pgen.1000641 (PMC2729386; doi:10.1371/journal.pgen.1000641)
Supplement: Text S1 — Supplemental information on details of AS analysis, its interpretation, and how it relates to previous data. (0.09 MB DOC) [file pgen.1000641.s010.doc]

**Text S1. Supplemental information on details of AS analysis, its interpretation and how it relates to previous data.**

**Phylogenetic trees of monomers**

Unrooted phylogenetic trees of monomers were constructed using different methods and sets of monomers. Generally the UPGMA method yielded better branch separation, and therefore these trees are shown in Figure 1 and described in the text. Same sets of monomers were also used to construct trees by neighbor-joining (NJ) method and the trees of very similar topology were obtained in all cases. Very similar NJ trees were obtained with the PHYLIP and MEGA4 program packages. As a rule, monomers sitting on one branch on a phylogenetic tree were grouped into one array and, conversely, the arrays marked in the same color mostly include the monomers that come from the same branch of the phylogenetic tree. However, a small number of “runaway monomers” breaking this rule were observed (shown in Figure 1). Most prominent runaway groups were noted in Figure 1B as follows: two groups of 8 H3 monomers each, in the H4 branch (4 orange + 4 lilac in one group and 8 lilac in the other; sublayer colors as described below and shown in Figure S3); 6 V1 monomers in the H1 branch (all from the bright green Xq sublayer, see below); and 10 M1 monomers from distal yellow Xq domain in the V1 branch. In Figure 1C the latter group is present as well and an additional group of 12 M1 monomers (3 from 17q and 9 from 8q) appears in V1 branch. In Figure 1A there are 2 M1 monomers in the V1 branch and two H1 monomers in H2 branch. A total number of runaway monomers in each tree did not exceed 2%.

The NJ trees obtained with the MEGA4 program were evaluated for reliability using interior branch test with 500 replicates. The test values for most major branches exceeded 95% and for all layer-defining branches were 89% or more (not shown). The trees were also constructed using various subsets of monomers for the purpose of mixing tests (e.g. every tenth red monomer instead of all of them). We also tested pericentromeric regions of each chromosome individually in separate runs and in combinations (e.g. X + 8). In all cases, the trees of very similar overall topology were obtained.

**Analysis of Xp AS**

Below we present the details of our monomeric comparisons referring both to our classification and to the designations (clades and types) used in previous Xp analysis [3,4].

***HOR border (clade 5)***. Monomeric typing reveals that the region about 10kb in length previously termed type 4 DXZ1 (part of clade 5) [4] is in fact formed by blue (SF5) monomers R1 (type B) and R2 (type A). The domain was originally defined as monomers that lacked the HOR structure and identifiable SF3 monomeric types, but appeared on the tree in the same clade with typical DXZ1 HOR, and was interpreted as DXZ1-derived poorly homogenized sequences. According to our new interpretation, clade 5 in [4] unites the SF5 family monomers (old AS of AB type) and the HOR monomers W1-W3 (type A) and W4-W5 (type B). SF5 is presumably a direct ancestor of all new SFs [26] including the SF3 that forms DXZ1 and is predictably closer to the HOR group of clades than to the clades of the older AS comprised of pure type A satellite. Clade 5 therefore, just unites all type AB satellite as opposed to type A in all other clades. The HOR/SF5 border is abrupt, with the zone of increased divergence being just a bit longer than two HOR units (Figure S1; HOR similarity plot). There is, therefore, no difference with an abrupt HOR/SF4 (yellow) junction in chromosome 17 described recently [24] and in this paper.

***171 bp vs. 172 bp satellite (clade 4)***. It appears that within the Xp AS array the monomer changes its length from 171 bp, characteristic of humans and great apes, to 172 bp, characteristic of monkeys. The HOR domain consists of 171bp-type W1-W5 monomers, the blue domain of SF5 R1 and R2 monomers which are also 171 bp long, and the yellow domain is represented by SF4 171 bp monomers of M1 type. On the border of the yellow and yellow-striped (V1) domain, monomer length switches to 172 bp now being structurally similar to S1 monomer of OWM and lacking the deletion in position 23 of the 172 bp monomer [1]. In the yellow domain all monomers are 171 bp. In the yellow-stripped domain, however, one-fourth of the monomers also have the deletion. These monomers are distributed irregularly within the domain and do not form a separate branch on the tree (not shown), which means that they do not represent a special subtype within V1 class of monomers or a fraction of M1 monomers interspersed in the V1 array. Thus, the deletion is likely a partially homogenized mutation in a transient state of fixation. The deletion is also present in about 3% of monomers in ancient layers, which is compatible with an overall occurrence of a deletion in these layers (not shown). In conclusion, the two subdivisions of clade 4 noted previously [4] (M1 and V1 in our terms) represent different monomeric types of different length, pass the compact residence test and therefore should be considered two different layers and two suprachromosomal families SF4 and SF6, respectively. The border between SF6 (yellow-striped) and SF4 (yellow) is abrupt (Figure S1).

***Olive-green domain contains dimeric AS (Clade 3)***. Clade 3 [4] consists of two branches, the members of which on closer inspection appear to alternate in an imperfect manner in the corresponding domain (not shown). Both monomers are of type A, thus representing the first, to our knowledge, case of dimeric, pure A-type AS in humans, reminiscent of 172 bp dimeric S1S2 A-type satellites of macaques [1]. Monomeric comparisons, however, did not reveal any particular sequence resemblance between H1H2 and S1S2 dimers (not shown). So, the two dimeric satellites have arisen independently and the S1S2 satellite is not present in human lineage and is specific to the baboon-macaque group.

***Number of Xp domains.*** Thus, the changes we have made in the interpretation of the Xp phylogenetic data are as follows: (i) clade 3 is made up of dimeric satellite, (ii) clade 4 divides into 2 distinct domains, SF6 (V1; 172 bp) and SF4 (M1; 171 bp), (iii) clade 5 divides into SF5 (R1R2) and HOR domains. Summarily, there are seven domains instead of five noted previously.

**Analysis of Xq AS**

Distinct domains were identified in Xq arm in a similar manner. Addition of Xq monomers to Xp tree in a mixing well test allowed us to identify all Xq domains with those of Xp, as depicted in Figure 2. Notably, the olive-green and grey domains are absent on Xq. The blue/yellow segment is repeated twice, but shows no evidence of being a duplication (not shown), probably resulting from another kind of rearrangement like inversion or additional insertion of a blue array in a yellow domain. Importantly, four of the seven Xp domains have counterparts on Xq with which they “mix well” on a phylogenetic tree. The succession of layers is roughly the same on both arms, thus showing a kind of tree growth ring symmetry. The HOR and 171/172 junctions on Xq are both abrupt.

**Chromosomes 8 and 17.**

The analysis of chromosomes 8 and 17 is presented in Figure 2. Only the layers previously identified on the X were found, and in a similar, partially symmetrical arrangement.

The partial analysis of chromosome 17 AS has been published recently [24]. To the 17q contig described there, we were able to add more BAC clones (AC141299.1, AC083828.3) and a contig NW_927772 anchoring it to the HOR domain and thus making the sequence of AS-containing 17q region nearly complete with the only gap within satellite III region. This arrangement is supported by BAC end analysis (not shown). The new portion contains W1-W5 14-mer HORs, a large stretch of SF5 (blue layer) and a small SF4 (yellow) domain. Our analysis confirms that the three red domains termed M1, M2 and M4 are closely related and “mix well” on the trees, and M3 (yellow-striped) is different [24]. The 17q 14-mer HOR, which we termed D17Z1-C, is represented by five 99% identical copies at the end of our 17q contig and gives numerous 99% identical hits in the WGS database. It is distinct from both the homogeneous 14-mer D17Z1-B HOR found on 17p side and D17Z1 HOR that forms current centromere and is represented by predominant 16-mer and polymorphic variants ranging in length from 15-mer to 12-mer [24]. The 3 types of HOR are 93.5% identical to each other. The adjacent organization of D17Z1 and D17Z1-C is supported by a working draft quality BAC AC084014, which contains both D17Z1-C and D17Z1. The size of D17Z1-C array on 17q is not known. The 17p HOR junction with old AS is abrupt as described previously [24] and the absence of SF5 layer on the p-arm is notable.

Unlike chromosomes 17 and X, which both have SF3 HORs, chromosome 8 contains SF2 HOR composed of D1-D2 dimers unrelated to W1-W5 pentamers ancestral to SF3. However, on chromosome 8 the HOR domain is surrounded by blue (R1R2), yellow (M1) and red (H3) domains, which mix well with respective species from chromosomes 17 and X (Figure 1C). Both HOR junctions in chromosome 8 are abrupt.

**New, old and ancient AS sequences**

The blue layer we describe in this paper corresponds to previously described SF5 [26] and consists of irregularly alternating R1 and R2 monomeric types. The yellow layer consensus sequence matches M1 monomeric type (171 bp long) identified previously [25] as a consensus of SF4. Therefore, the majority of sequences initially used to define SF4 actually belonged to the yellow layer. However, particular sequences classed as SF4 in previous publications were defined just as monomeric AS more closely related to M1 consensus (AJ130755) than to the monomeric types characteristic of SFs 1, 2, 3, and 5. The older 172 bp monomers would also match this criterion. So, in fact, M1 and SF4 were used as collective names for all monomeric AS. For instance, having revisited the sequences classed as SF4 in our review [1] published in 2001, we found that although the majority of sequences belonged to the yellow layer, yellow-striped (V1) and even ancient sequences were present (not shown). We propose to keep the names SF4 and M1 for the yellow layer and treat the older layers as separate SFs.

Phylogenetic trees shown in Figure 1 demonstrate that V1 (yellow-striped), M1 (yellow) and R1R2 (blue) monomers form three subclades sitting on a common stem. This branch is well separated from others and unites both 171 and 172 bp monomers. The common stem corresponds to the common ancestor of all apes as opposed to NWM and OWM. Statistical analysis of similarity relationships (not shown) also demonstrates that important distinction lies not between 171 bp and 172 bp monomers, but between yellow-striped (V1) and the older layers. It can be illustrated by the pronounced difference in relative similarity to overall AS consensus [26] derived from 12 known 171 bp monomeric types (ALPHA-ALL), plotted on Figure S1. There, a clear step, roughly from 50% to 70% relative similarity, can be seen between olive-green and yellow-striped layers on Xp, and between red and yellow-striped on Xq. This distinction is also reflected in the plot of identity to DXZ1 HOR shown on the same picture, and on phylogenetic trees. Hence, in Table 1, the 172 bp yellow-striped monomeric type was termed V1 and classed with the old families and the older 172 bp monomer was termed type H (to mark human lineage as opposed to lower primate-specific S1 through S5 types [1]). Phylogenetic concession of new, old and ancient AS would thus correspond to African apes, apes and monkeys, respectively. Ancient and old AS monomers can be preliminarily discriminated automatically by virtue of their relative similarity (rs) to ALPHA-ALL (rs <0.6 for ancient and rs> 0.6 for old; see legend to Figure S1). For now, we do not want to assign the suprachromosomal family names to the ancient families, other than naming their monomeric types. Both discovery of few other ancient layers in other chromosomes, and re-interpretation of the sublayers are possible, so it seems better to wait till more complete information is available, and the names more or less reflecting the chronological order of the families can be assigned.

**Genome-wide presence of various layers**

The genome-wide distribution of SF5 (blue) AS sequences has already been previously demonstrated [1]. Our additional studies indicate that the order of layers: new – blue (SF5) – yellow (SF4) is conserved on most chromosomes (see Table S6 for some examples). In order to analyze the distribution of other major domains identified here and collect samples for L1 dating, we performed a genome-wide scanning by the probes representing three AS layers: yellow-striped (V1; SF6), olive-green (H1H2) and grey (H4) sequences. After obtaining lists of highly identical sequences the samples were preliminarily sorted into the following categories: (i) old AS (ALPHA-ALL identity) with the presence of a large proportion of 172 bp monomers would represent yellow-striped layer, (ii) ancient (ALPHA-ALL identity) and dimeric (dot matrix) AS would represent olive-green layer, and (iii) ancient non-dimeric AS would represent red + grey layers. Sorted monomers were further tested using cladistic analysis for mixing well on the trees with our established layers from chromosome X. Using this approach we were able to confirm the presence of each layer on a number of chromosomes (see Table S2), thus proving the genome-wide distribution of the major AS domains.

**Differentiation within the layers**

As it was mentioned in “Results”, some layers and respective clades demonstrate further differentiation and could perhaps be divided into a number of sublayers. Notably, three clades in the red branch designated by letters B, C and J in Figure 1C demonstrate only a limited degree of mixing on phylogenetic trees and tend to occupy different subdomains, shown as orange, red and lilac in Figures S1 and S2. However, clade G in Figure 1C is composed of a mixture of monomers of all 3 colors and therefore these sublayers do not formally pass the compact residence test. Similarly, the yellow-striped domain on Xq could be divided in two distinct parts (shown on Figures S1 and S2 as yellow-striped and bright green). In all cases these subdivisions are closely related. For clarity, in this work we treated clades and domains as separate layers only if they showed no significant mixing. However, the assumption that separate layers never mix may not truly reflect the real recombination pattern. For instance, some mixing may occur on the borders as a function of time and/or because some kind of short-distance residual homogenization may operate even in the dead centromeres after the layers have been formed. In any case, occasional gene conversions should result in some degree of mixing that can be dependent on sequence identity in respective layers, the physical distance between them and on the length of time during which the recombination events have been accumulating. Below we discuss the alternative scenarios of sublayer formation in more detail.

The lilac and orange domains mainly appear in an asymmetrical manner. Therefore, the lilac – orange – red division may be explained as three different centromeric sequences (three separate layers in our terms; interchromosomal transfer/amplification scenario) or as three stages of evolution of the red layer (three sublayers of the red layer; segment cut off scenario). In the first case, one has to assume multiple events that created asymmetry on chromosomes 8 and X. In the second, to believe that two pairs of events took part simultaneously at two different stages of the red layer evolution. Namely, the lilac domain on Xq represents a part excluded from the red layer at the same time as the red array on chromosome 17 went dead and was frozen at the lilac stage. Also, the orange domains on Xp and 8q have been cut off the respective red arrays at the same time. It should be noted that the layer pattern revealed in this paper reconciles the differences between two earlier attempts of AS analysis [23,24]. Contrary to the previous finding [23] that monomeric AS from certain chromosomes mixed well with monomeric AS from other chromosomes, it was found that monomeric AS taken from distal regions of Xp, 8p and both arms of chromosome 17 formed chromosome-specific subclades that did not intermix significantly [24]. It is clear now that, in this analysis, parts of grey (Xp) and red (8p) domains where compared to chromosome 17 yellow-striped and lilac layers. However, as expected, some mixing of red and lilac monomers was noted, as seven red 8p monomers were interspersed in chromosome 17 lilac cluster (see Fig. 4 in [24]). Such limited mixing was interpreted as errors of phylogenetic inference, rather than evidence of monomer exchange. In our analysis, the 8p red domain mixed well with the X red domains, chromosome 17 lilac domains mixed well with Xq lilac domain, and 8q orange domains mixed well with Xp orange array. Consistent, albeit limited, mixing of orange, red and lilac sublayers was also notable.

One more instance of sublayers observed in our work, the bright green subdivision in the yellow-striped domain on Xq (see Figure S2), may be explained as a part of the yellow-striped domain excluded by inversion, as the whole bright green segment is inverted relative to the bulk of the domain (Figure S1A).

As mentioned in “Discussion”, it seems likely that the more symmetrical proper layers could be mainly formed via an “interchromosomal transfer/amplification” mechanism and the less symmetrical sublayers are more likely to be formed by a “segment cut off” mechanism. However, this impression may change upon mapping of other chromosomes, if multiple cases of symmetrical lilac and orange layers will be observed. It has to be noted that centromeric layers would serve as a kind of “phylogenetic record” regardless of their mechanism of origin. Either way they all are discrete and chronologically ordered, getting younger towards current centromere. As expected, there is an intra-array divergence gradient lilac> orange> red and all three sublayers are present in various primates (data not shown).

**L1 dating**

In this analysis the independently established age scale of L1 families, which were active sequentially [31], is used to grade the age of AS target sequences.

L1 repeats were scored in various AS layers and in the genomes of various primates, such as lemur (*Lemur catta*), tarsier (*Tarsius syrichta*), NWM (*Callithrix jacchus*), OWMs (African green monkey, macaque, baboon) and apes (gibbon, orangutan, gorilla and chimpanzee). About 3 Mb of BAC clones were scored for each species. Only the elements which could be classed to an individual L1 family were scored. When clusters of L1s are present in AS, only the elements with at least one end in AS (i.e. the ones which are likely to have integrated directly into AS) were scored. For each layer, the oldest major L1 family shared with humans was determined. The results are shown in Table S3 and summarized in Table 2.

Khan and co-authors [31] have established that the genome copy numbers for L1 families from PA2 to PA7 are comparable, with PA2, PA6 and PA10 being about twice less abundant than the others. The major peaks of activity of these families did not occur simultaneously, but succeeded each other with only a moderate overlap. They have estimated the age of all primate L1 subfamilies and scored them in the genomes of lemur, baboon and chimpanzee. Their results corresponded pretty well to observations of this study (Table S3) with one exception: They did not find any PA5 in the baboon genome. To make sure of that point we performed some additional scoring and analysis of PA5 in baboons and proved beyond doubt that it was present (not shown). The neutral mutation rates in L1 were calculated as 0.126% per million years based on human/orangutan and 0.216% based on human/lemur comparisons [31]. We used the higher mutation rate for our estimates of AS age, as it better matched the generally accepted age of the primate taxa [32] and our data on L1 occurrence in various primates (shown in Table 2). It also corresponded well to neutral mutation rate 0.20% - 0.22% derived from comparisons of various ancestral repetitive sequences in cattle, dog and humans [44].

**Current centromeres of primates**

Our study was aimed at dead satellite layers and we did not focus on examination of current centromeric AS repeats in primates. So, any hints on composition of the current primate centromeres come either from literature or from circumstantial evidence found in the course of this study. Combination of homogeneity and high copy number can be used as a criterion for identification of the current centromeric sequence, because, so far, homogeneous (0%-5% divergent) high copy number AS repeats were found only in functioning centromeres. Such satellites of OWM and NWM were reviewed earlier [1]. In *M. mulatta* (OWM) and *C. jacchus* (NWM), used as examples in Table 2, they are represented by monomeric types S1S2 and S3S4, respectively. Numerous homogenous R1R2 repeats highly identical to ti885841334 (see Table S6) with dimeric and tetrameric periodicities are found in orangutan. Therefore, the current centromeres in at least some orangutan chromosomes may be formed by the blue (SF5) AS layer. As no clearly orthologous sequences are found in humans, these HOR-like repeats are likely the result of SF5 evolution in orangutan ancestors after the orangutan - African apes split. Similarly, a great abundance of “yellow-like” primate-specific AS is observed in gibbon, including homogenous trimeric HOR-like sequences highly identical to ti2054969403 (see Table S6). It indicates that current gibbon centromeres are likely to be formed by yellow-derived AS. “Yellow-like”, in this context, means old, 171 bp long AS, which is closer to monomeric type M1 than to any other human monomeric types. Phylogenetic analysis of HOR-like sequences previously observed in gibbon [22] was consistent with this conclusion. On phylogenetic trees these monomers formed a separate branch which clustered, but did not mix with new AS from chimpanzee and man, and was well separated from the human ancient AS (see Fig. 6A in [22]). As there are no type B monomers in gibbon ([37] and our analysis), and hence there are no new families and SF5, such clustering is likely to indicate 171 bp yellow AS. These data indicate that the current gibbon and orangutan centromeres are formed by the yellow SF4-derived and the blue SF5-derived AS, respectively, as opposed to the new AS families in the gorilla – chimpanzee – human group [1]. This conclusion, however, needs to be confirmed in a detailed study, which would become possible with further progress of chromosome-mapped contigs in these primates.

**Divergence analysis in AS domains**

The estimates of mutation rates and of the age of AS layers were obtained using a formula for similarity between two sequences diverged from the common ancestor [43] *s*=1/4+3/4e-8*^mt^*^/3^, where “s” is similarity, “m” is mutation rate and “t” is the time since divergence. The actual mutation rates, estimated from the orthologous human – primate comparisons shown in Table S5, were 0.17%, 0.19% and 0.21% per million years, for gorilla, orangutan and *M. mulatta*, respectively. For age calculation based on intra-array divergence, shown in Table 4, a rate of 0.2% and the mean “within layer” similarity figures from Table 3 were used.

The faster-than-expected accumulation of mutations indicated by the discrepancy between intra-array and interspecies divergence in the dead layers, leads to a suggestion that homogeneous AS arrays may be subject to a mutation rate few times higher than that in the dead divergent arrays. The high rate of evolution was previously noted for homogeneous AS arrays as compared to the dead AS. Rudd and co-authors [24] have compared the interspecies differences for the grey array with the divergence of homogenized X-specific HOR arrays, in human and chimpanzee and demonstrated that homogenized AS arrays evolve considerably faster (1.3% versus 5% divergence). One explanation would be that the fast accumulation of mutations is somehow a consequence of an intense homogenization process. On the other hand, it might be possible that the rapid evolution is due to a higher mutation rate, which is a peculiar property of homogeneous AS arrays perhaps dependent on their perfect identity and tandem organization. It is kept at bay by homogenization, which does not allow any divergence to accumulate, while the array remains a functional centromere and hosts a kinetochore. Once KARM moves to a new centromere the hypermutability is given a free reign and mutations occur at a very high rate until the near perfect identity of repeats is ruined and mutation rate gradually subsides to a normal level.

Notably, the substitution rate at the 3’ UTR of L1 retroposon source genes (ORF2) is much higher than usual, presumably due to the reduced fidelity of reverse transcriptase and RNA polymerase, which both lack a proofreading activity [45]. In the case of AS, it may be due to a low fidelity DNA repair synthesis caused by replication problems. Homogeneous monomeric satellite array presents a replication conundrum as there will be no origins at all in the vast arrays or a replication origin in almost every monomer. In African green monkey a typical monomer which forms homogeneous arrays on all chromosomes and comprise about a half of the genome, can serve as an origin of replication [34]. However, similarly high mutation rate is observed in non-repeated yeast centromeres [46].
